# Brachytherapy for the Conservative Treatment of Female Peri-Urethral Carcinoma

**DOI:** 10.3390/cancers14030845

**Published:** 2022-02-08

**Authors:** Mickaël Andraud, Manon Kissel, Roger Sun, Elie Rassy, Sophie Espenel, Samir Achkar, Philippe Morice, Christine Haie-Meder, Sébastien Gouy, Cyrus Chargari

**Affiliations:** 1 Department of Radiation Oncology, Gustave Roussy Cancer Campus, 94800 Villejuif, France; mickael.andraud@aphp.fr (M.A.); manon.kissel@curie.fr (M.K.); roger.sun@gustaveroussy.fr (R.S.); sophie.espenel@gustaveroussy.fr (S.E.); samir.achkar@gustaveroussy.fr (S.A.); christine.haiemeder@gustaveroussy.fr (C.H.-M.); 2 Department of Medical Oncology, Gustave Roussy Cancer Campus, 94800 Villejuif, France; elie.rassy@gustaveroussy.fr; 3 Department of Surgery, Gustave Roussy Cancer Campus, 94800 Villejuif, France; philippe.morice@gustaveroussy.fr (P.M.); sebastien.gouy@gustaveroussy.fr (S.G.)

**Keywords:** vaginal cancer, cervical cancer, brachytherapy, radiotherapy, image-guided radiotherapy, radiation oncology

## Abstract

**Simple Summary:**

Para-urethral gynecological tumors pose significant therapeutic challenges due to the expected morbidity of surgery, especially when the tumor is close to the urethra. There is little data to guide management. Most of the available data have focused solely on the treatment of primary vaginal tumors, and few publications provide guidance for the treatment of vaginal recurrences of other gynecologic cancers (e.g., cervix, endometrium). We report on the feasibility of interstitial brachytherapy, as an exclusive treatment combined with external radiotherapy, for the conservative treatment of para-urethral tumors, with high local control probability.

**Abstract:**

**Introduction:** Peri-urethral cancers (PUC) are rare tumors. Brachytherapy (BT), either monotherapy or combined with radiation therapy, is a preferred treatment option to spare the morbidity of surgery and achieve organ preservation. We report, to the best of our knowledge, the largest experience of brachytherapy among women with PUC. **Patients and Methods:** This is a retrospective review of the medical records of female patients with PUC who underwent low- or pulse-dose-rate BT with or without external beam radiotherapy at Gustave Roussy between 1990 and 2018. Patients were categorized according to the treatment intention into a primary and recurrent group. The Kaplan–Meier method was used for survival analysis, and the Cox proportional-hazard model was used for univariate analysis. Brachythewharapy-related adverse events were reported according to Common Terminology Criteria for Adverse Events version 4. **Results:** We identified 44 patients with PUC who underwent BT. Of the 44 patients, 22 had primary tumors and 22 had recurrent tumors. Histologies were mainly adenocarcinoma (*n* = 20) and squamous cell carcinoma (*n* = 14). The median prescribed dose was 60 Gy for the 24 patients treated with BT alone and 20 Gy (IQ range: 15–56.25 Gy) for the 20 patients treated with BT in combination with EBRT. With a median follow-up of 21.5 months (range 7.5–60.8), a total of six patients experienced local relapse (17.5%). The 2-year overall survival probability was 63% (95%CI: 49.2–81.4%). The most common toxicities were acute genito-urinary grade 1–2 toxicities. At the last follow-up, four patients experienced focal necrosis. **Conclusions:** In this cohort of women with PUC undergoing BT, we observed an 80% probability of local control with acceptable morbidity. Though survival was poor, with high metastatic relapse probability, BT was useful to focally escalate the dose and optimize local control in the context of an organ sparing strategy.

## 1. Introduction

Para-urethral cancers (PUC) are rare but aggressive tumors that account for less than one percent of genitourinary malignancies [1,2]. These tumors are located in the tissues around the urethral ostium and the tissues surrounding the perineal urethra. Para-urethral cancers include a spectrum of diseases with various histologies. They mainly extend from primary urethral and vaginal cancers but can also represent a metastatic site for various malignancies, including endometrial and cervical cancer. Moreover, squamous cell carcinoma and adenocarcinomas represent the predominant carcinomas, though numerous histologies can be found [3,4,5,6].

The local treatment of patients with PUC presents therapeutic challenges because of the increased risk of complications using these local interventions. Surgical approaches require wide resection margins, leading in this situation to significant morbidity and poor outcomes in terms of organ preservation; an anterior pelvectomy is frequently the only possible surgical option. Radiation therapy is an alternative treatment that has the advantage of preserving organ function. Brachytherapy potentially has an important role in the management of para-urethral tumors, given its ability to focally escalate the dose, which is especially relevant in radioresistant tumors such as urethral carcinoma, and to minimize organs at risk. It has been shown for primary vaginal tumors that a brachytherapy boost was associated with a significant benefit in survival, compared to external radiotherapy only [7,8,9,10,11,12,13]. However, the risk of vaginal necrosis and fistula is to be taken into consideration and interstitial brachytherapy procedures are challenging in this rare situation. In this study, we describe the treatment techniques and report the efficacy and safety outcomes of brachytherapy among patients with PUC. 

## 2. Materials and Methods

### 2.1. Patient Selection and Data Collection

We retrospectively reviewed the medical records of adult patients with biopsy-proved PUC that underwent with brachytherapy at Gustave Roussy between 1990 and 2018. In this study, we included patients with PUC extending to less than one centimeter from the urethral ostium, including patients treated for a para-urethral recurrence from cervical or endometrial cancer. Patients with missing follow up, treated for a vulvar cancer, and PUC occurring in a previously irradiated field were excluded; those with a history of previous pelvic irradiation were, however, included if the vaginal tumor was located below the radiotherapy fields. Details concerning patient characteristics, pathology, and previous treatments were collected. Patients were assigned to the primary group and recurrence group according to the clinical situation: treatment of a primary PUC or treatment of a PUC metastatic recurrence from gynecological cancer. Toxicities were scored according to the Common Terminology Criteria for Adverse Events version 4. Acute toxicities were defined as toxicities occurring less than six months after treatment completion. Late complications were defined as any complication occurring after at least six months of follow-up. This retrospective study was conducted in accordance with ethical standards and was approved by a local ethics committee.

### 2.2. Therapeutic Indication

All patients underwent a comprehensive staging, including an abdominopelvic magnetic resonance imaging (MRI) and a computed tomography (CT) of the abdomen and pelvis or 158-fluorodeoxyglucose Positrons Emission Tomography (18-FDG PET/CT) to check for regional lymph nodes and distant metastases. An urethrocystocopy was not systematically performed but was guided by symptoms and imaging to rule out intraluminal extension and to obtain an exhaustive tumor cartography. Subsequently, the treatment indications were approved after a multidisciplinary discussion, taking into account previous treatments and data from primary staging.

Schematically, the treatment decisions were as follows: 1. Patients with a primary vaginal cancer received brachytherapy boost following whole pelvis external beam radiotherapy with or without concurrent chemotherapy. In addition, exclusive brachytherapy was discussed among patients with a localized urethral tumor and without regional lymph node extension. 2. Patients with para-urethral recurrences underwent brachytherapy boost after external beam radiotherapy if the patient had no history of pelvic lymph node irradiation or BT as an exclusive local treatment if the patient had isolated para-urethral relapse and a history of previous external beam radiotherapy. Locoregional lymph node areas were always included in external beam radiotherapy volumes for primary vaginal tumors. In the case of para-urethral relapse, regional lymph nodes were included if the patient had not been previously irradiated, especially for patients with synchronous lymph node relapse. Concurrent chemotherapy, weekly cisplatin at 40 mg/m^2^, could be delivered concomitantly with external beam radiotherapy in patients with adequate renal and hematological function. An additional cycle of chemotherapy was discussed at the time of brachytherapy in order to deliver a total of at least 5 cycles of chemotherapy (total dose: 200 mg/m^2^ cisplatin). If cisplatin was contraindicated, weekly carboplatin Area Under Curve 2 was proposed.

### 2.3. Brachytherapy Procedure

**Figure 1:**
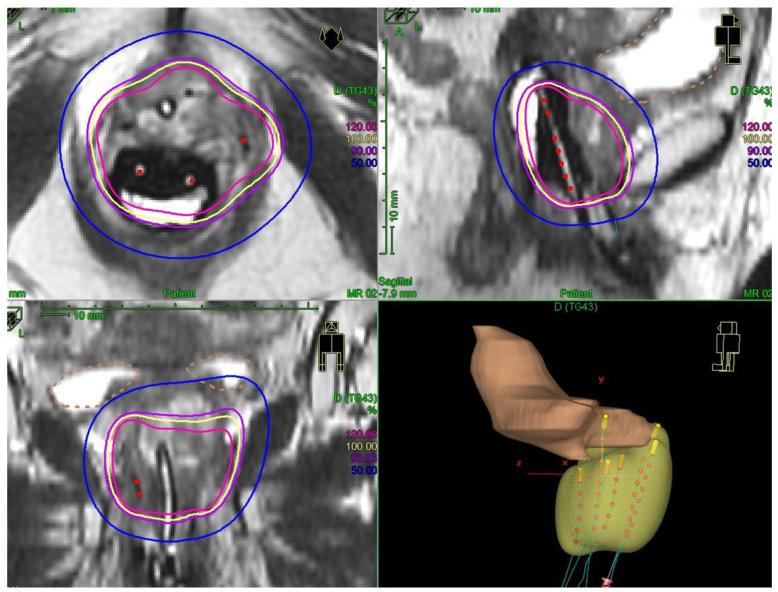
Example of image-guided pulse-dose-rate treatment for a primary para-urethral carcinoma. Patient was treated with pulse-dose-rate brachytherapy boost, delivering 15 Gy in pulse-dose-rate brachytherapy. In yellow is shown the 100% isodose. A vaginal mold applicator with anterior catheters was used and three interstitial needles were implanted to encompass the urethra.

For all patients, a personalized vaginal mold applicator was built from a vaginal impression to adapt the number and positions of the brachytherapy catheters according to the tumor shape and extent. Brachytherapy implant was performed under general anesthesia, allowing a careful gynecological examination to decide on the implant geometry. Transperineal free-hand interstitial needles were implanted for patients with a tumor thickness of more than 7 mm. At the time of implant, fiducial seeds could be implanted to visualize tumor extents on radiographs or CT. Before 2014, treatments were delivered through continuous low-dose-rate (LDR) brachytherapy. Thereafter, treatments were delivered through pulse-dose-rate (PDR) technology. For patients treated at the time of LDR brachytherapy, brachytherapy treatment planning was based on 2D radiographs. For patients treated with PDR, 3D imaging (MRI and/or CT) was performed with an applicator in place. Clinical target volumes were defined according to clinical and radiological findings. For patients treated with brachytherapy as a boost, a brachytherapy dose of 15–20 Gy was prescribed, with the objective to deliver a total dose of at least 60 Gy to initial sites of disease (clinical target volume = CTV), taking into account the contribution of external beam radiotherapy and with dose escalation at the level of residual disease (residual gross tumor volume at the time of brachytherapy = GTV). For patients treated with exclusive brachytherapy, the objective was to deliver at least 70 Gy to the macroscopic tumor (GTV) and 60 Gy to the tumor plus a 3D anatomical margin of 5–10 mm (CTV). No dose constraint was applied for the urethra that could be part of the target volume. For applications performed before 2014, Cesium-137 was used for intracavitary applications and Iridium-192 wires were used as interstitial sources. After 2014, pulse-dose rate brachytherapy was used and stepping source Iridium-192 was used for both intracavitary and interstitial treatments. An example of 3D treatment planning for a periurethral adenocarcinoma is shown in Figure 1.

### 2.4. Follow Up

The first assessment of tumor response relied on a gynecological examination and a pelvic MRI performed at 6–8 weeks following brachytherapy. Thereafter, patients were examined every 3 months for 2 years, then every 6 months for 3 years. Systematic MRI was not performed in the follow-up and was guided by clinical symptoms and signs. The first sites of relapse were examined and classified as local (primary tumor site), regional (pelvic and/or inguinal lymph nodes) or distant (metastases).

### 2.5. Statistical Analysis

Descriptive statistics were used to describe patient and tumor characteristics. Survival rates were calculated from treatment completion to the occurrence of the studied event. The median progression-free survival (PFS) and overall survival (OS) were estimated using the Kaplan–Meier method; two-year OS, PFS, survival without distant failure and local control were estimated with a 95% confidence interval (95% CI). Local control was defined as the absence of local relapse, defined as a relapse occurring in the lower half of the vagina, urethra, bladder neck or in the vulva. PFS was defined from the date of brachytherapy completion to the date of progression or death and OS was defined from the day of brachytherapy completion to the date of death. Survival without metastatic event was defined from the day of brachytherapy completion to the date of metastatic progression or death. Patients who did not progress and were alive at the time of the last follow-up were censored at the time they were last seen for PFS and OS analyses, respectively. Complete remission was defined as a disappearance of all signs of cancer as per clinical and radiological examination at the first post-treatment assessment. Hazard ratios and associated 95% confidence intervals were calculated with the use of the Cox proportional hazard model for univariate analysis. We did not perform a multivariate analysis given the small sample size. Statistical analyses were performed using R version 4.0.4 (R Core Team (2021). R: A language and environment for statistical computing. R Foundation for Statistical Computing, Vienna, Austria. URL: https://www.R-project.org/ accessed on 23 August 2021).

## 3. Results

### 3.1. Patient Characteristics

A total of 44 patients eligible for this analysis were identified; they had a median age of 65 years (range: 54–78). The main histologies were adenocarcinomas (46%), including 10 patients with metachronous para-urethral distant relapse of uterine endometrioid adenocarcinoma. Fourteen patients (32%) had squamous cell carcinomas, including eight patients with primary vaginal tumor and five patients with metachronous para-urethral relapse from gynecological tumor (five from cervical cancer and one from vaginal cancer), and the median tumor size was 20 mm (range: 2.5–30) at the time of treatment. Tumor extension was considered to be local in 34 patients (77.3%) and locoregional in 10 patients with lymph node metastases to the groin, pelvis and para-aortic area in six (14%), five (11%), and three (7%) patients, respectively. Among the patients with regional extension, two patients had pelvic and para-aortic lymph node metastases and one patient had inguinal, pelvic and para-aortic lymph node extension. The patients’ characteristics are detailed in Table 1.

### 3.2. Patterns of Care

Among the 22 patients in the primary group, 16 patients received external radiotherapy (including 10 with concurrent chemotherapy) before brachytherapy. Six patients were treated with exclusive brachytherapy. These six patients presented a localized urethral tumor, with tumor size ≤ 30 mm, and had no sign of regional lymph node extension. 

Among the 22 patients in the recurrence group, 18 were treated with exclusive brachytherapy, three had external radiotherapy before brachytherapy (with chemotherapy in one patient with synchronous pelvic lymph node relapse), and one had exclusive brachytherapy of para-urethral tumor site, associated with external beam radiotherapy to treat bilateral groins. The patient had macroscopic lymph node disease in the groins and a history of pelvic irradiation for bladder cancer contra-indicating the delivery of pelvic irradiation.

### 3.3. Brachytherapy Characteristics

Brachytherapy characteristics are detailed in Table 2, for the whole cohort and according to the treatment group. Eighteen patients had brachytherapy treatment planned on 2D radiographs, and 26 patients treated with PDR brachytherapy had treatment planned on CT with target volumes delineated using a fusion MRI. The recurrence group underwent exclusive brachytherapy more frequently (86% vs. 27%, *p* < 0.01) and with a higher dose (60 Gy vs. 20 Gy, *p* < 0.01). There was no significant difference in the use of interstitial needles between the two groups (91 vs. 77%, *p* = 0.412). Among the patients treated with image-guided brachytherapy, 15 had 3D dose/volume data available. Median D2cc (minimal dose delivered to the most irradiated 2cc parts of the organ, calculated in equivalent doses per 2 Gy fractions (EQD2) with alpha/beta value = 3 Gy for organs at risk and 10 Gy for tumors, half-time repair of 1.5 h for DNA damages) were 48.9 Gy_EQD2_ (range: 16–64 Gy_EQD2_) for rectum, and 58.3 Gy_EQD2_ (range: 9–64 Gy_EQD2_) for bladder. The minimal dose delivered to 90% (D_90_) of the GTV was 70 Gy_EQD2_ (range: 57–129 Gy_EQD2_) and minimal D_90_ of the CTV was 54 Gy_EQD2_ (range: 51–109 Gy_EQD2_). The overall treatment time was 5.9 days for patients treated with exclusive BT (calculated from first to last brachytherapy pulse) and was 53.1 days for patients treated with brachytherapy boost (calculated from first external radiotherapy fraction to last brachytherapy pulse).

### 3.4. Efficacy Outcomes

**Figure 2:**
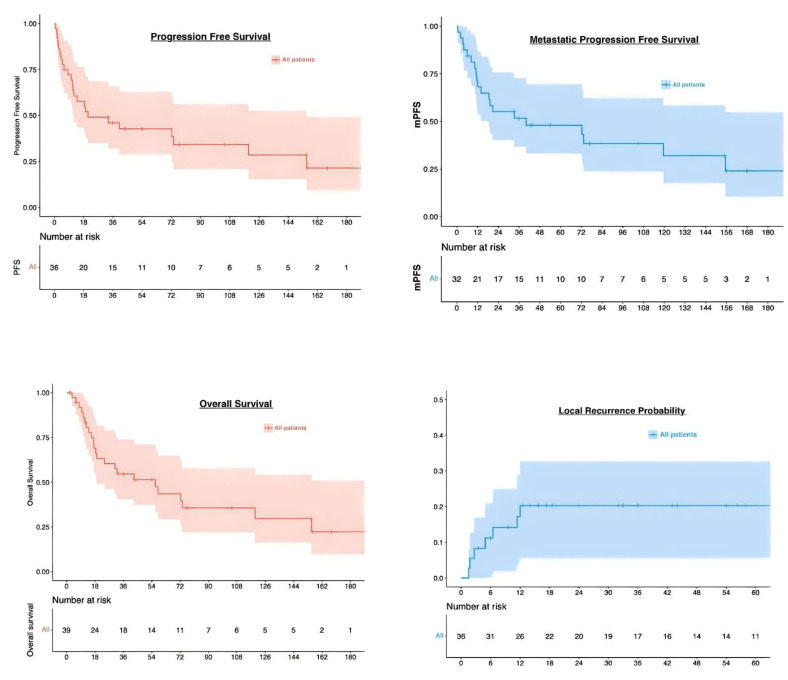
Survival and local recurrence probability curves.

At the first post-treatment assessment, complete remission was achieved among 17/22 patients (77%) and 16/22 patients (72%) in the primary and recurrent groups, respectively (not significant). After a median follow up of 21.5 months, the local control probability was 80% in the whole cohort (95%CI: 67.3–94.4%). At two years, PFS, the probability of survival without metastatic event, and OS were 49% (95%CI 35.0–68.7%), 55% (95%CI: 40.2–75.7%), and 63% (95%CI: 49.2–81.4%), respectively (Figure 2). Ten patients (23%) had distant failure in the course of their disease. When examining patient outcomes according to the brachytherapy technique used (image-guided PDR vs. LDR), there was a trend toward better OS among patients treated with PDR, though significance was not reached (HR: 0.44, 95 % CI: 0.19–1; *p* = 0.055). At two years, OS probability was significantly poorer among patients with local relapse (HR: 5.2, 95 % CI: [1.6–18]; *p* = 0.017).

### 3.5. Safety Outcomes

The acute brachytherapy-related adverse events were mainly grades 1–2 (*n* = 27, 61%): grade 1–2 urinary symptoms were reported in 19 patients (43%) and a grade 2 mucositis was reported in 15 patients (34%). Only one patient presented acute severe complication (grade 3 vaginal toxicity). Most late toxicities were low grade; grade 1–2 urinary incontinence was reported in seven patients (16%). Toxicities are described in Table 3. Two patients required dilatation of brachytherapy-related urethral stenosis. No late rectal morbidity was reported. Two patients had late grade 2 vaginal stenosis. Both had received exclusive brachytherapy. Four patients had late grade 3 complications, with focal vaginal necrosis. Two had received LDR brachytherapy boost, one had PDR brachytherapy boost, and one had received exclusive LDR brachytherapy. Among these four patients, two had an irradiated volume> 150 cm^3^ (153 cm^3^ and 182 cm^3^). 

## 4. Discussion

Radiation therapy is the standard treatment for patients with para-urethral tumors in the setting of an organ-sparing strategy to avoid the mutilation of anterior pelvectomy. This study reports the largest cohort of patients conservatively treated in the same center for a primary or recurrent para-urethral tumor with brachytherapy.

For patients with primary vaginal tumors, treatment is mainly based on retrospective series, showing that external radiotherapy followed with brachytherapy boost, concomitant with chemotherapy, is the reference treatment [7,12]. The place of brachytherapy in the treatment is essential. A study based on the analysis of the SEER database reported a doubling of the median OS when brachytherapy boost was delivered following external radiotherapy for vaginal tumors [14]. Recently, a retrospective study reported that pelvic control was obtained in approximately 75% (range: 39–92%) of patients, with 5-year OS around 58% (range: 34–86%) [13]. By analogy with cervical cancer, the place of adaptive image-guided brachytherapy is increasing, with retrospective series suggesting a benefit in terms of local control and treatment-related morbidity [15,16]. Such series, however, did not specifically examine the outcome of patients with para-urethral tumors and mixed patients with various tumor topographies and, therefore, distinct patterns of treatment.

For patients with para-urethral recurrences from gynecological tumors, very scarce data are available. The two main series were published by Greven et al. in 1998 and Sharma et al. in 2016 [17,18]. Greven et al. reported on a series of 10 patients with para-urethral recurrence of endometrial cancer (seven patients) or cervical cancer (three patients). Five of them had previously received pelvic radiotherapy. The tumor size varied from 1 to 6 cm. Six patients were treated with a combination of external radiotherapy and LDR brachytherapy and four patients with exclusive LDR brachytherapy. The mean total dose received was 40–65 Gy for previously irradiated patients and 65–75 Gy for non-previously irradiated patients. With a median follow-up of 34 months (range: 1–64 months), four patients were alive with no sign of local or metastatic recurrence at the end of the follow-up. One patient recurred distantly after 60 months and received chemotherapy, one patient died of pelvic recurrence and four patients died from metastatic progression. The authors observed only one severe toxicity with a rectovaginal fistula occurring at 21 months from brachytherapy [17]. Sharma et al. reported on a cohort of 10 patients with para-urethral tumors, five patients with primary tumors, and five patients having a para-urethral tumor treated by brachytherapy with or without external radiotherapy. The tumor size varied from 1.5 to 5 cm. Brachytherapy implantation was carried out using a free-hand interstitial procedure. Five patients received a combination of external radiotherapy (range: 36–50 Gy) and brachytherapy (range: 18–21 Gy), and five others received exclusive brachytherapy (range: 37–42 Gy). With a median follow-up of 25 months (4–74 months), 6/10 patients were disease free. Seven patients presented with acute grade 3 mucositis. No late grade 3–4 toxicity was reported. Two patients had an inguinal recurrence; one had local recurrence and one had both local and inguinal recurrence. All three patients with inguinal recurrences had been treated with exclusive brachytherapy. The local control rate at the last follow-up was 80% [18].

Our study, including 50% primary para-urethral tumors and 50% para-urethral relapse, aims at providing additional data on this rare clinical presentation and examining the possibility to perform a conservative treatment. We report a local control probability of 80% at 2 years, comparable to previously described data. With a higher number of patients, we show that good local control can be achieved with brachytherapy either for primary tumors or for the treatment of para-urethral recurrences of pelvic tumors. OS at 2 years was 63%, also in line with previously described data, with distant failure being the main pattern of relapse. In our series, the distant failure probability was reported in 45% of patients, highlighting the need for systemic intensification in these patients. In addition, 25% of patients had relapse from endometrial cancer, and at the time of study molecular profiling was not done on a routine basis. This may contribute to the high frequency of distant failures in these patients. The prognosis was poorer than in cervical cancer or than reported in other series of primary vaginal tumors. First, local control was lower, probably because dose escalation was limited given the higher risk of necrosis compared to other tumors located in the upper part of the vagina or developed from the cervix. Second, almost half of the patients had a previous history of pelvic radiotherapy, precluding delivering a prophylactic pelvic lymph node irradiation, possibly explaining the high frequency of distant events. Finally, half of the patients were referred for locoregional relapse. Although the safety profile was acceptable, we observed four cases of focal vaginal necrosis, predominantly in the group treated before 3D imaging and stepping source technology use. Among the two patients with available information concerning the treated volume, the brachytherapy V100% was very large in both of them (>150 cm^3^). Dose and volume have a major impact on severe toxicities for brachytherapy treatments, though data in this specific clinical situation are still uncertain and our study does not allow drawing definitive conclusions on dose/volume parameters, given the low number of patients with available 3D dosimetric data [19,20]. No urethral dose constraint was followed. One specificity for para-urethral tumors is that the urethra can be part of the target volume (if initially involved) but is also an organ at risk. Contrary to prostate cancers, where urethra is usually not involved, there can be very close contact between the urethra and gynecological tumors, and therefore the urethra could be included as part of CTV (Figure 1). A prospective EMBRAVE study will collect clinical and dosimetric data for patients with primary vaginal tumors. This study should provide meaningful information in terms of dose/effects relationship for dosimetric optimization guidance. 

Our study has several limitations. The collection is retrospective and monocentric, with the inclusion of patients with various primary tumors treated with different technologies over the last three decades. This time span leads to selection bias, with patients probably not staged in an equivalent way with the advancement of diagnostic tools. In addition, there was a major evolution of brachytherapy, especially with the shift from 2D to 3D treatments, and from low-dose rate to remote afterloading technology, allowing for better dose-optimization processes. These technological improvements may have a major impact in terms of oncological and functional outcomes. In addition, the majority of patients were referred for brachytherapy only, after having received their primary treatment in another center. In spite of these limitations, we observed a local control of 80% with brachytherapy within most of the cases with moderate toxicities, suggesting the feasibility of this conservative approach to avoid the morbidity of an anterior pelvectomy in patients with para-urethral tumors. The finding that most relapses are distant encourages the development of novel strategies based on immunomodulation or systemic intensification.

## 5. Conclusions

In this cohort of LDR/PDR BT for para-urethral primary or recurrent cancer, we observed a probability of local control of 80% with acceptable morbidity. Survival was, however, poor, with high metastatic relapse probability. There is an increasing place for 3D image guidance in these tumors that should be referred to expert centers for modern brachytherapy procedures as part of organ conservative strategies. Further dose–volume constraints still need to be identified to guide treatment planning. 

## Figures and Tables

**Table 1:** Tumors’ characteristics and treatment clinical indications.

Characteristics		*n*
**Number of Patients**		44
**Age (median [IQR])**		65 [54, 78]
**Histology (%)**	Adenocarcinoma	20 (46)
	Squamous cell carcinoma	14 (32)
	Urothelial carcinoma	5 (11)
	Other *	6 (11)
**Patients groups (%)**	Primitive tumor	22 (50)
	Recurrent tumor	22 (50)
**If relapse, primary tumor site (%)**	Cervix	7 (16)
	Uterus	11 (25)
	Uterine sarcoma	1 (2)
	Bladder	2 (5)
	Vulva	1 (2)
	Primitive	22 (50)
**Brachytherapy indication (%)**	Post-radiotherapy boost	19 (43)
	Exclusive brachytherapy	25 (57)
**Surgery (%)**	No	31 (71)
	Yes	13 (29)
**Previous irradiation (%)**	No	26 (60)
	Yes	18 (40)
**Size of tumor in mm (median [IQR])**		20.00 [2.5, 30.0]
**Nodal status (%)**	Node negative	34 (77)
	Node positive	10 (23)

IQR: interquartile range; * others: neuroendocrine carcinoma (*n* = 1); pagetoid carcinoma (*n* = 1); sarcomatoid carcinoma (*n* = 1); adenoid cystic carcinoma (*n* = 1); leiomyosarcoma (*n* = 1).

**Table 2:** Treatment characteristics according to patient groups.

Characteristics		Primary Treatment	Recurrence	*p*
**Number of patients**		22	22	
**Brachytherapy modality (%)**	Boost	16 (73)	3 (14)	< 0.001
	Exclusive	6 (27)	19 (86)	
**EBRT technique (%)**	No	6 (27)	19 (86)	0.001
	3D	11 (50)	2 (9)	
	IMRT	5 (23)	1 (5)	
**Interstitial implantation (%)**	No	2 (9)	5 (23)	0.412
	Yes	20 (91)	17 (77)	
**Dose in Gy (median [IQR])**		20.00 [15.00, 56.25]	60.00 [60.00, 60.00]	0.005
**Brachytherapy modality (%)**	LDR	8 (36)	10 (46)	0.760
	PDR	14 (64)	12 (54)	
**v100% in cm^3^ (median [IQR])**	LDR	51.87 [23.64, 97.57]	63.10 [56.00, 182.14]	0.361
	PDR	47.91 [18.95, 82.81]	59.20 [38.15, 63.62]	0.897

BT: brachytherapy; EBRT: external beam radiotherapy; LDR: low-dose-rate brachytherapy; PDR: pulse-dose-rate brachytherapy; V100%: body volume receiving 100% of the prescription isodose.

**Table 3:** Toxicities according to the Common Terminology Criteria for Adverse Events version 4.

		Grade	*n* (%)
**Acute toxicity**	Urinary symptoms	1–2	19 (43)
		3–4	0 (0)
	Mucositis	1–2	15 (35)
		3–4	1 (2.3)
	Rectal inflammation	1–2	1 (2.3)
		3–4	0 (0)
**Late toxicity**	Necrosis	0	20 (45.5)
		1	4 (9.1)
	Incontinence	1–2	7 (16)
		3–4	0 (0)
	Vaginal stenosis	1–2	3 (7)
		3–4	1 (2.3)

## Data Availability

Data are not available.
